# National Hotel Disease

**Published:** 1857-08

**Authors:** 


*National Hotel disease.*—At a regular meeting of the New York academy of medicine at which Dr. Mott presided, Dr. James Wyne of Baltimore read a paper upon the National Hotel disease. Upon examination of the Hygeinic condition of the house, he says: an opening was found in the south-west corner of the cellar, through which a strong current of fetid gas was escaping from the sewer. Both the Committee appointed to investigate the causes of the complaint, and Medical gentlemen in attendance at the Hotel, concurred in the belief that the evil was due to noxious exhalations from this source. Reported to the New York Journal of Medicine

☞ A Dr. Coggswell "Down East" has been advertising Extensively in the newspapers, particularly in *Massachasetts*, an *Antiphlogistic Salt*, which its *discoverer* says, will cure nearly "all the ills that flesh is heir to;" and near half a hundred editors, have borne their testimony in the most unequivocal manner, to its having cured, under their observation, nearly as many different kinds of disease —*Peninsular Journal of Medicine.*

